# Diseases of the Intracranial Vessels in Children

**Published:** 1891-03-28

**Authors:** 


### DISEASES OF THE INTRACRANIAL VESSELS IN CHILDREN.

The intracranial vessels in youth seem at first sight to be placed under conditions singularly favourable to their safety and well-being. Free from the degenerative changes of later life, they are protected from external injuries by the skull, whose open sutures make fracture difficult, and which in its growing state rapidly recovers from severe violence. Still, evidence is daily accumulating of the number and importance of the diseases found in these vessels. The extreme compression of parturition often breaks them down; they, almost alone of the whole body, suffer from aneurysms in youth; they are blocked or broken down by syphilis; thrombosis within them is held on good grounds to be the cause of infantile hemiplegia; and they act as distributors to the tissues about them of at least four of the most fatal septic poisons to which infant mortality is due. The tubercular virus spreads, as is well known, from the basal vessels to set up its special form of meningitis; the pneumonic poison is carried by them to produce the epidemic meningitis so fatal on the Continent, or to cause the head complications of ordinary pneumonia; the poison of ulcerative endocarditis, is carried along them to form new foci of disease; and, lastly, suppurative otitis pours into them pyæmic matter in abundance to form fatal abscesses and thrombi.

It has of late been shown that a large number of cases of idiocy, paralysis, and especially of spastic paralysis, are due to difficult parturition, especially when the head is born last. The brain substance itself at this stage of life is very tolerant of injury, but the minute vessels under extreme pressure often give way, and large quantities of blood are poured out over the vertex, compressing especially the cortical centres for the legs and trunk. The difficulties of diagnosis are very great. It may be months or almost years before anything amiss in the movements of the limbs can be made out, and then treatment is of little avail, except gymnastic exercises, by which, as Gowers points out, voluntary co-ordination of movements may be gradually acquired in many instances. Modern brain surgery is beginning to find a field for itself in these cases, and when a large effusion can be discovered at an early date, it seems possible to remove it and to give the convolutions a chance to recover before convulsions and spastic paralysis set in. Hence the great importance of early and minute examination of infants when there is reason to suspect such hæmorrhages.

Infantile hemiplegia, with its sequelæ of shortened limbs and choreic movements, whether due to a thrombosis of these vessels or to a primary myelitis, is, unfortunately, less open to any possibility of treatment other than educational.

The formation of aneurysms in the larger vessels is of very great interest. They appear to be just as frequent in the decade from ten to twenty as in any equal period up to sixty, although of extreme rarity in the rest of the body. On the other hand, miliary aneurisms, so frequent in the degenerated brain vessels of later life, are unknown in childhood. Dr. Church pointed out in 1870 the connection of these large aneurysms with heart disease and emboli, and gave a list of thirteen cases in early life. He held that an embolus which caused partial obstruction produced dilatation of the vessel wall behind it, which gradually gave way from failure of nutrition. Ponfick, from a series of cases of his own, argued that for the production of aneurysm it was necessary that the embolus should be hard and pointed, that the surrounding tissues should be soft and yielding, such as the brain or spleen, that the place of lodgment should be just beyond a branch, and that the obstruction as a rule should be partial. He thought that the hard embolus would injure the wall, and the injured spot would gradually yield and at length form an aneurysm. His explanation, though suited to his own cases, certainly does not seem applicable to many others. The emboli are not always hard calcareous fragments detached from the valves of the heart, and the aneurysm does not always occur beyond a bifurcation. Holmes has laid down that aneurysms are sometimes caused by an embolus, and result from dilatation on the proximal side of the obstruction. But this can only take place under special circumstances, since the space above is usually filled with thrombus, and, as Dr. Goodheart remarked, the surgeon does not usually look for an aneurysm after ligature of the femoral. That embolism is a cause of aneurysm is perfectly clear from the position of the embolus found in some of these cases of intracranial aneurysm in the young, where, too, no degeneration of the walls exists. Besides, most of them occur where heart disease is or has been present. It is most probable that, for the formation of an aneurysm, the obstruction, unless it occur just beyond a branch, must be incomplete, so that no thrombus be formed. In the circle of Willis, indeed, owing to the peculiarities of the circulation there is little chance of a clot forming if the obstruction is complete. It is noticeable that, common as are aneurysms and hæmorrhages from the smaller vessels of the brain in later life, aneurysms upon them in childhood are practically unknown.

We think that Dr. Goodheart has indicated an important factor in these cases by pointing out that they generally follow ulcerative endocarditis. He argued that the clot contained septic matter and induced an arteritis at the spot where it lodged, thus weakening the wall to a fatal degree. It is certain that such endocarditis has existed in a large number of cases, and where the other factors are present, such a secondary arteritis seems to afford the best explanation of the aneurysm.

Aneurysms occur most frequently in the middle cerebral, next in the basilar, and next in the internal carotid. Gowers notes that it has been denied that an embolus could lodge in the basilar, but he has himself described and figured in the first volume of Brain an embolus in that place. The symptoms are too often insufficient for diagnosis. Headache, vertigo, strabismus, and other paralyses, and very rarely a murmur have been noticed, but frequently there is little warning before rupture occurs, followed more or less gradually by convulsions and death. A basilar aneurysm has been thought to produce an occipital headache, but in a recent case of our own we noted that the headache was distinctly frontal. More or less recently, ligature of the carotid or vertebrals has been tried as a remedial measure as well as treatment by iodide of potash and rest, and cases of spontaneous cure have certainly occurred.

There are other instances of aneurysm in youth where no heart disease can be traced, as in the case referred to above, and Gowers has pointed out that hereditary syphilis is present in many of them, and is the probable cause of the failure of the vessel. Some form or other of syphilitic affection of the vessels is not very rare, and the difficulty is perhaps to explain why it occurs so seldom.

The subject of tubercular meningitis, and of the form coming with pneumonia, is closely allied to that of the distribution of pyæmic particles in endocarditis. The poisons in the former diseases enter, however, through the lung, and from the minute size of the particles to which they are attached may penetrate to very small vessels in the brain. Possibly septic particles do sometimes pass through the lungs on a voyage from a more distant source.

Recent investigations on Fraenckel's pneumococcus tend to show that it becomes the active agent in pneumonia when the conditions of the patient are favourable to it; that it travels to the capillaries of the brain, producing the delirium and other meningeal symptoms so frequently seen, and that in other cases, rarer in England, it seems to leave the lungs almost untouched, passing at once to the vessels of the brain and cord, causing what is known as cerebro-spinal meningitis, and often becoming virulently infectious. In pneumonia its activity is exerted chiefly on the lungs and slightly on the brain, while in meningitis the stress falls on the intracranial vessels. Still, in either disease both regions are more or less affected.

In all these disorders we see the poison once introduced into the system finds a convenient spot for multiplication in the tissues lying round the vessels of the brain. Our chief effort, then, must be to stop them at their entrance, before the heart or lung mischief can develop, since if they are carried on to the intracranial vessels we have very little chance of attacking them in the present state of bacterial knowledge. Another common scourge of childhood, purulent otitis, often sets up pyæmic infection in the meningeal vessels, though more frequently it brings on thrombosis of a vein or sinus. Here, early remedial measures are of even greater value, for when taken early the infection of the vessels can be generally prevented. It must also be remembered that cases of diarrhœa in children sometimes end fatally from thrombosis of the longitudinal sinus.

It does not seem possible as yet to explain why the vessels in childhood are so extremely liable to certain of the diseases we have mentioned, such as tubercular meningitis, but our review of them has shown at least the vast importance of detection of the early stages of the complaint and the protection of the intracranial vessels from septic invasion.

